# Post-mortem examination of high mortality in patients with heart failure and atrial fibrillation

**DOI:** 10.1186/s12916-022-02533-8

**Published:** 2022-10-05

**Authors:** Otilia Țica, Ovidiu Țica, Karina V. Bunting, Joseph deBono, Georgios V. Gkoutos, Mircea I. Popescu, Dipak Kotecha

**Affiliations:** 1 Institute of Cardiovascular Sciences, Medical School, University of Birmingham, Vincent Drive, Birmingham, B15 2TT UK; 2 Cardiology Department, Emergency County Clinical Hospital of Oradea, Gheorghe Doja street, No 65, 410165 Oradea, Romania; 3 Pathology Department, Emergency County Clinical Hospital of Oradea, Gheorghe Doja street, no 65, 410165 Oradea, Romania; 4 Queen Elizabeth Hospital, University Hospitals Birmingham NHS Foundation Trust, Mindelsohn Way, Birmingham, B15 2GW UK; 5 Institute of Cancer and Genomic Sciences, University of Birmingham, Birmingham, B15 2TT UK; 6 Health Data Research (HDR)-UK Midlands, Institute of Translational Medicine, B15 2GW Birmingham, UK

**Keywords:** Heart failure, Atrial fibrillation, Mortality, Autopsy, Post-mortem

## Abstract

**Background:**

The prevalence of combined heart failure (HF) and atrial fibrillation (AF) is rising, and these patients suffer from high rates of mortality. This study aims to provide robust data on factors associated with death, uniquely supported by post-mortem examination.

**Methods:**

A retrospective cohort study of hospitalized adults with a clinical diagnosis of HF and AF at a tertiary centre in Romania between 2014 and 2017. A standardized post-mortem examination was performed where death occurred within 24 h of admission, when the cause of death was not clear or by physician request. National records were used to collect mortality data, subsequently categorized and analysed as HF-related death, vascular death and non-cardiovascular death using Cox proportional hazards regression.

**Results:**

A total of 1009 consecutive patients with a mean age of 73 ± 11 years, 47% women, NYHA class 3.0 ± 0.9, left ventricular ejection fraction (LVEF) 40.1 ± 11.0% and 100% anticoagulated were followed up for 1.5 ± 0.9 years. A total of 291 (29%) died, with post-mortems performed on 186 (64%). Baseline factors associated with mortality were dependent on the cause of death. HF-related death in 136 (47%) was associated with higher NYHA class (hazard ratio [HR] 2.45 per one class increase, 95% CI 1.73–3.46; *p* < 0.001) and lower LVEF (0.95 per 1% increase, 0.93–0.97; *p* < 0.001). Vascular death occurred in 75 (26%) and was associated with hypertension (HR 2.83, 1.36–5.90; *p* = 0.005) and higher LVEF (1.08 per 1% increase, 1.05–1.11; *p* < 0.001). Non-cardiovascular death in 80 (28%) was associated with clinical obesity (HR 2.20, 1.21–4.00; *p* = 0.010) and higher LVEF (1.10 per 1% increase, 1.06–1.13; *p* < 0.001). Across all causes, there was no relationship between mortality and AF type (*p* = 0.77), HF type (*p* = 0.85) or LVEF (*p* = 0.58).

**Conclusions:**

Supported by post-mortem data, the cause of death in HF and AF patients is heterogeneous, and the relationships with typical markers of mortality are critically dependent on the mode of death. The poor prognosis in this group demands further attention to improve management beyond anticoagulation.

**Graphical Abstract:**

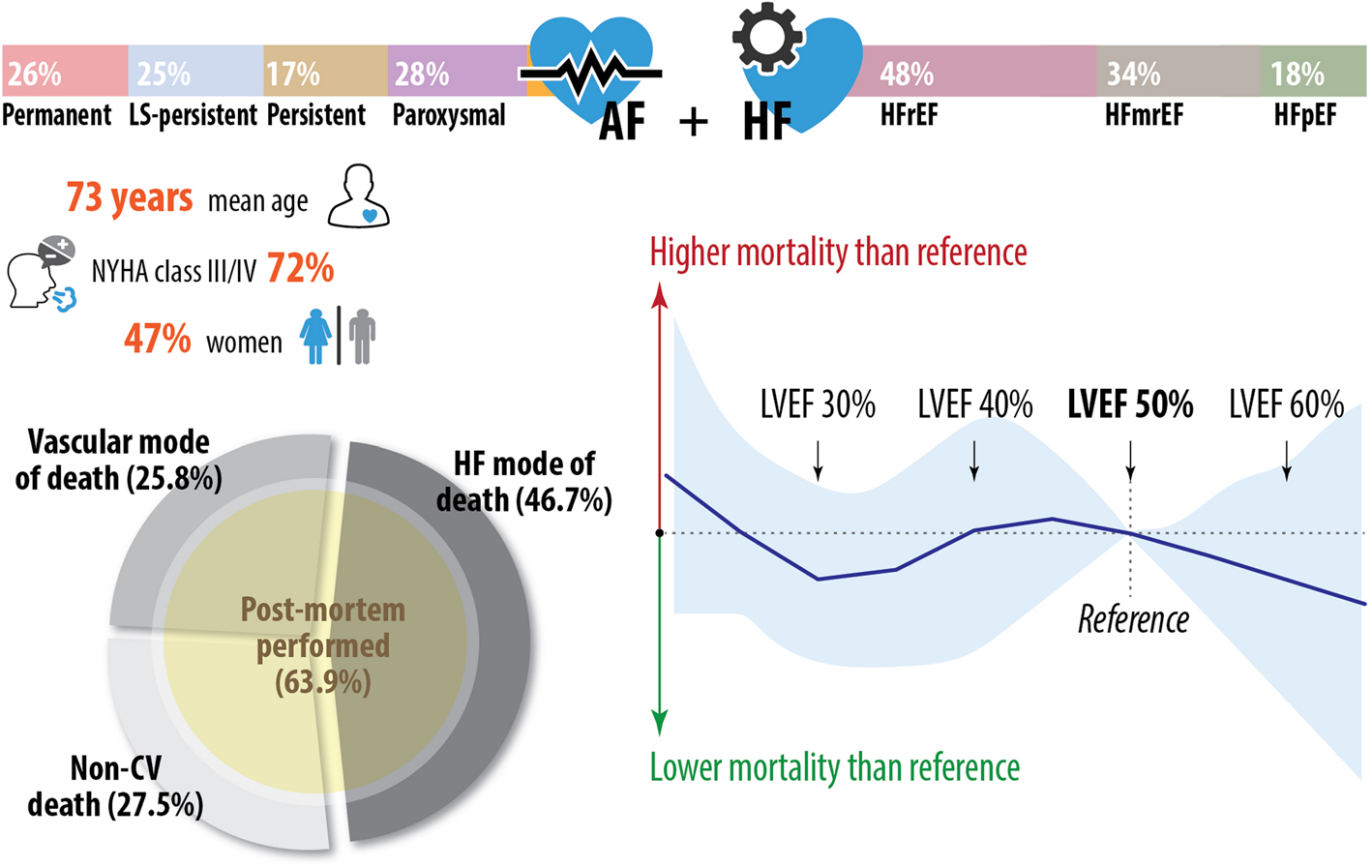

**Supplementary Information:**

The online version contains supplementary material available at 10.1186/s12916-022-02533-8.

## Background

Heart failure (HF) and atrial fibrillation (AF) are two of the most frequent cardiovascular conditions encountered in daily practice. Although they share similar risk factors with interconnected pathophysiology, they can also exacerbate one another leading to a worse prognosis [1–3]. Patients that share both conditions are typically multi-morbid, more often frail, and have worse symptoms. The combination of HF and AF poses an increasing burden on healthcare systems due to their hospitalization and treatment-related costs [4, 5]. Management strategies have typically focused on anticoagulation to prevent stroke and thromboembolism; however, the most common adverse event in these patients is actually mortality. Trials and observational cohorts have confirmed high mortality rates for HF and AF with both reduced ejection fraction (HFrEF) and preserved ejection fraction (HFpEF) [6, 7].

In clinical practice, we currently lack an understanding of what patient factors are associated with mortality in patients with both HF and AF and whether attention to these factors could reduce the high burden of adverse events. The cause of death is often frequently misrepresented in clinical practice [8], and attributed to death certificates using generic statements, such as heart failure-related death or sudden death. This imprecise categorization of death may be masking important associations and interactions [9]. For example, hypertension is likely to be a determinant of death related to vascular causes, such as myocardial infarction and stroke, but could confound the association between age, left ventricular ejection fraction (LVEF) and other causes of death, such as decompensated HF. In this study, we hypothesized that a better understanding of the cause of death, supplemented by post-mortem examination, could allow for differentiation of specific modes of death, reveal underlying etiological factors, and inform the clinical management of patients with concomitant HF and AF.

## Methods

This retrospective observational study included consecutive adult patients hospitalized at the Emergency County Clinical Hospital of Oradea (SCJUO), Romania, between January 2014 and December 2017. The study was conducted in accordance with the ethical principles set out in the Helsinki Declaration and Recommendations for Good Clinical Practice and was approved by the SCJUO Ethics Committee (32,926/2017) without the need for individual patient consent. Additional file 1: Online Methods provides further details on the study approach. This follows the CODE-EHR best practice framework for the use of structured electronic healthcare records in clinical research [10]. This study meets all five of the CODE-EHR minimum framework standards (see Additional file 2 for the checklist).

### Patient population

Patients were included if they presented with HF and AF to the cardiology department or transferred from another department at SCJUO or other local hospitals. Heart failure was categorized using common clinical classification into HFrEF (LVEF < 40%), mildly-reduced/intermediate (LVEF 40–49%) and HFpEF (LVEF ≥ 50%; see Additional file 1: Table S1 for definitions) [11]. LVEF was based on echocardiography performed by accredited cardiologists; the most recently available data were used, either performed during the admission or within the previous 6 months. The diagnoses of AF from the clinical team were retrieved and categorized using accepted definitions (Additional file 1: Table S2) [12]. To avoid missing data, patients without a complete follow-up available were excluded (for example, four patients who lived outside the region) and also patients whose death was due to a violent cause and required investigation by forensic specialists.

### Clinical factors, tests and comorbidities

Clinical factors were determined at the time of admission from the patient’s electronic medical record. Information on participants was collected within a database system separate from any clinical databases. Data collected included demographic information, associated clinical evaluations and comorbidities and treatments received during the hospital admission and at discharge. Laboratory data and drug information were used to confirm specific comorbidities, in addition to complementary investigations, such as cardiac and non-cardiac imaging. The diagnosis of ischemic heart disease was based on the patient’s history of significant coronary heart disease by coronary angiography or based on chest pain associated with an increased level of changes in cardiac markers (troponin I or highly sensitive troponin I) and/or echocardiographic changes consistent with ischemia or a positive non-invasive stress test. Hypertension was defined based on clinical history, use of antihypertensive therapy, a systolic blood pressure ≥ 140 mmHg or a diastolic blood pressure ≥ 90 mmHg. Diabetes was defined based on serum glucose levels greater than 126 mg/dL, glycosylated haemoglobin values greater than 6.5% or the use of oral antidiabetics or insulin. Ischaemic or haemorrhagic stroke was certified by a computed tomography performed during hospitalization in patients with neurological deficits. The CHA_2_DS_2_-VASc thromboembolic risk score was calculated with points for heart failure, hypertension, age ≥ 75 years [double], diabetes mellitus, previous thromboembolism [double], vascular disease, age 65–74 years and female gender. The HAS-BLED bleeding risk score was calculated with points for uncontrolled hypertension, creatinine > 2.2 mg/dL or dialysis, cirrhosis or elevated liver tests, prior stroke, prior bleeding, labile control of warfarin, age > 65 years, medication predisposing to bleeding and excess alcohol intake. N-terminal pro-B-type natriuretic peptide (NTproBNP) levels were analysed using an accredited and calibrated Pathfast assay (Pathfast devices, Mitsubishi Chemical Europe), with a detection range of 15–30,000 pg/mL.

### Assessment of mortality

Deaths were extracted from the hospital database electronic medical record, as well as from the Unique Integrated Computer System of the Social Health Insurance in Romania (SIUI). The primary outcome was mortality in-hospital or during the follow-up period, categorized into heart failure-related death, vascular death and non-cardiovascular death.

Confirmation of events required documentary evidence supporting the diagnosis (for example, a death certificate, pathologist notes and autopsy report). Post-mortem autopsies were performed where death occurred within 24 h of admission, when the cause of death was not immediately clear or when requested by the attending physician, unless the next of kin refused consent. Post-mortems were performed according to a standardized protocol (Additional file 1: Online Methods). At the time that autopsies were performed, pathologists had full access to the patient charts, the clinical medical history and the results of any additional investigations, in order to fully correlate post-mortem findings (see example in Additional file 1: Fig. S1).

### Statistical analysis

Values are presented as median ± interquartile range (IQR; 25th to 75th centiles) or percentage. Group comparisons were assessed with the Kruskal–Wallis non-parametric analysis of variance test. Mortality was analysed with Cox regression models, presented as hazard ratios (HR) with associated 95% confidence intervals (CI). Multivariate models were prespecified to include age, gender, New York Heart Association (NYHA) class, LVEF, type of AF (paroxysmal versus non-paroxysmal), obesity (body mass index ≥ 30 kg/m^2^), coronary artery disease, hypertension, chronic obstructive pulmonary disease and diabetes. For multivariate analysis, patients with new-onset AF were included in the paroxysmal category, regardless of therapy, with sensitivity analyses excluding these patients finding no material difference in results. As natriuretic peptides are not specific to HF and are also prognostic in patients with coronary disease [13], NTproBNP was kept out of multivariate models so as not to obscure other associations. All models were also adjusted for medical therapy at baseline (statins, renin–angiotensin–aldosterone antagonists, beta-blockers, diuretics, amiodarone and digoxin therapy). Interactions were assessed with likelihood ratio testing, and the proportional hazards assumption in Cox models was confirmed using Schoenfeld residuals. In those that died, separate Cox models were generated to compare the differences in associations between heart failure-related death, vascular death and non-cardiovascular death. Kaplan–Meier plots were used to present the pooled, unadjusted data and number at risk, with the log-rank test of equality used to compare the groups. LVEF was modelled using a restricted cubic spline analysis in the Cox model.

A two-tailed *p*-value of < 0.05 was considered statistically significant. Where multiple comparisons were performed, an adjusted *p*-value was used. Analyses used complete case data as the amount of missing data was small (no imputation performed). Statistical analysis was performed with Stata (version 14.2, StataCorp LP, TX).

## Results

A total of 1009 consecutive patients with HF and AF were hospitalized in the study period, with a median age of 72.8 ± 10.5 years and 476 (47.2%) women. The mean LVEF was 40.1 ± 11.0%, with HFrEF (LVEF < 40%) present in 487 (48.3%), mildly reduced/intermediate HF (LVEF 40–49%) in 342 (33.9%) and HFpEF (LVEF ≥ 50%) in 180 (17.8%). The mean NYHA class was 3.0 ± 0.9, with 727 (72.0%) in class III or IV indicating severe or disabling symptoms. New-onset AF was seen in only 33 patients (3.3%), with the remainder evenly distributed between paroxysmal, persistent, long-standing persistent and permanent AF. All patients (100%) were anticoagulated with either vitamin K antagonists or a direct oral anticoagulant (dabigatran, rivaroxaban or apixaban) prior to or during admission.

### All-cause mortality

Over a mean follow-up of 1.5 ± 0.9 years, 291 patients (28.9%) died, of which 186 had a post-mortem performed (63.9% of patients who died; see Additional file 1: Table S3 for the characteristics of those undergoing autopsy). Baseline characteristics and comorbidities, comparing those alive and dead at follow-up, are presented in Table 1. There was no difference in either the CHA_2_DS_2_-VASc thromboembolism score or the HAS-BLED bleeding risk score. The median NTproBNP value on admission for those that died was 8710 pg/mL (IQR 4871–20,430); Additional file 1: Fig. S2. In-patient ventricular arrhythmias were documented in 57 patients (5.7%); 21 subsequently died (36.8%) which was not significantly different to those without ventricular arrhythmias (28.4%; *p* = 0.18).

**Table 1:** Baseline characteristics

Characteristics		Died during follow-up (*n* = 291)	Alive at the end of follow-up (*n* = 718)	*p*-value
Age, mean % ± SD		73.2 ± 9.0	72.6 ± 11.0	0.65
Women, *n* (%)		133 (45.7%)	343 (47.8%)	0.55
Background (urban vs rural setting), *n* (%)		133 (45.7%)	370 (51.5%)	0.09
NYHA class	Mean ± SD	3.0 ± 0.9	3.0 ± 0.9	0.25
	Class I, *n* (%)	23 (7.9%)	35 (4.9%)	
	Class II, *n* (%)	53 (18.2%)	171 (23.8%)	
	Class III, *n* (%)	110 (37.8%)	289 (40.3%)	
	Class IV, *n* (%)	105 (36.1%)	223 (31.1%)	
LVEF	Mean % ± SD	40.0 ± 11.0	40.1 ± 11.1	0.52
	< 40%, *n* (%)	146 (50.2%)	341 (47.5%)	
	40–49%, *n* (%)	98 (33.7%)	244 (34.0%)	
	≥ 50%, *n* (%)	47 (16.2%)	133 (18.5%)	
AF type, *n* (%)	New onset, *n* (%)	9 (3.1%)	24 (3.3%)	0.45
	Paroxysmal, *n* (%)	85 (30.1%)	202 (29.1%)	
	Persistent, *n* (%)	52 (18.4%)	122 (17.6%)	
	Long-standing persistent, *n* (%)	76 (26.9%)	178 (25.6%)	
	Permanent, *n* (%)	69 (24.5%)	192 (27.7%)	
Coronary artery disease, *n* (%)		213 (73.2%)	331 (46.1%)	< 0.001
Hypertension, *n* (%)		204 (70.1%)	378 (52.6%)	< 0.001
Chronic obstructive pulmonary disease, *n* (%)		88 (30.2%)	263 (36.6%)	0.054
Diabetes mellitus, *n* (%)		98 (33.7%)	195 (27.2%)	0.039^a^
Chronic kidney disease, *n* (%)		94 (32.3%)	215 (29.9%)	0.46
Prior stroke or transient ischemic attack, *n* (%)		45 (15.5%)	116 (16.2%)	0.79
Obesity (clinical diagnosis), *n* (%)		92 (31.6%)	175 (24.4%)	0.018^a^
CHA_2_DS_2_-VAS_C_ score, mean ± SD		5.1 ± 1.3	5.2 ± 1.3	0.26
HAS-BLED score, mean ± SD		3.0 ± 1.6	3.1 ± 1.4	0.11
ACE inhibitor or ARB, *n* (%)		225 (77.3%)	514 (71.6%)	0.063
Beta-blockers, *n* (%)		176 (60.5%)	377 (52.5%)	0.021
Diuretics or MRA, *n* (%)		242 (83.2%)	587 (81.8%)	0.60
Amiodarone, *n* (%)		111 (38.1%)	228 (31.8%)	0.052

*ACEi* angiotensin-converting enzyme inhibitors, *AF* atrial fibrillation, *ARB* angiotensin receptor blocker, *CHA*_*2*_*DS*_*2*_*-VAS*_*C*_ risk score for thromboembolism in AF, *HAS-BLED* risk score for bleeding in AF, *IQR* interquartile range, *LVEF* left ventricular ejection fraction, *MRA* mineralocorticoid receptor antagonist, *NYHA* New York Heart Association, *SD* standard deviation

^a^No longer significant when accounting for multiple testing

**Fig. 1:**
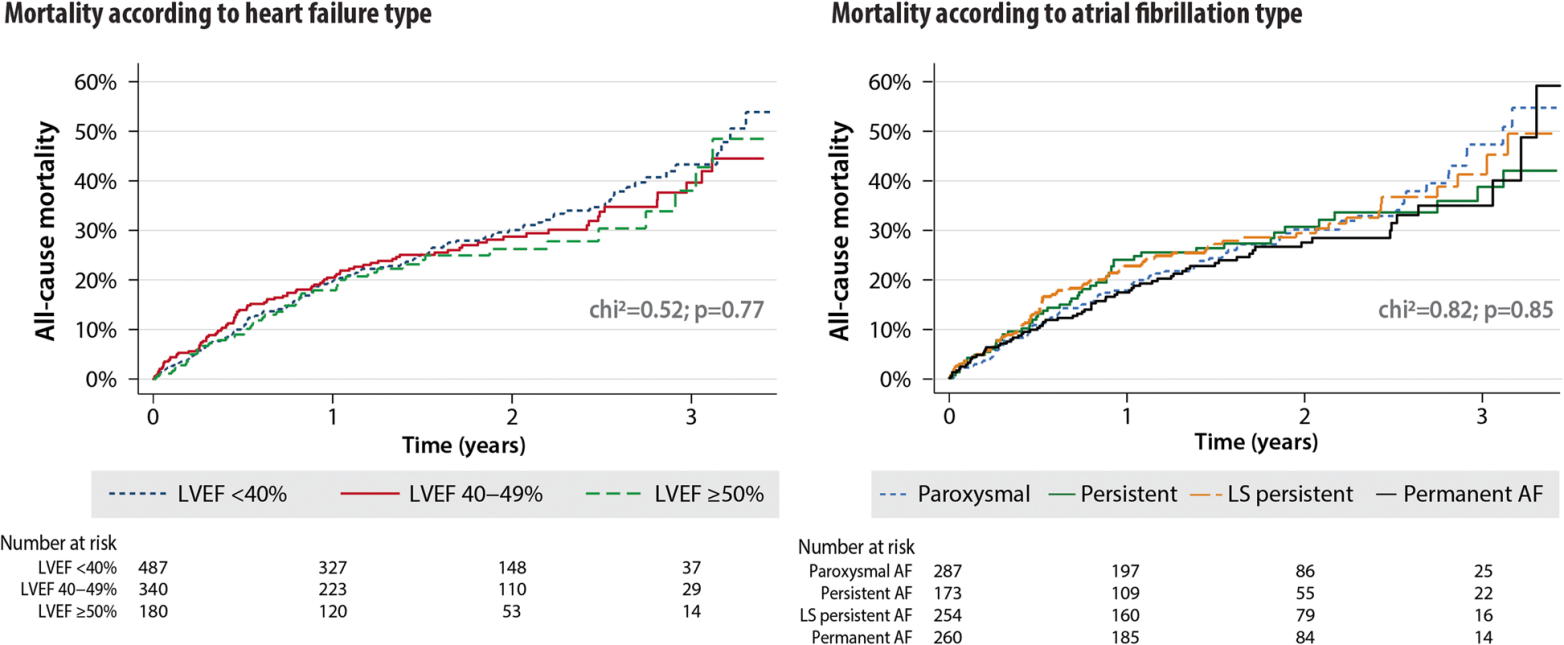
All-cause mortality by HF category and AF type. Kaplan–Meier plots for all-cause mortality according to the category of heart failure based on LVEF (left) and type of atrial fibrillation based on temporal pattern (right). AF, atrial fibrillation; LS, long-standing; LVEF, left ventricular ejection fraction

**Fig. 2:**
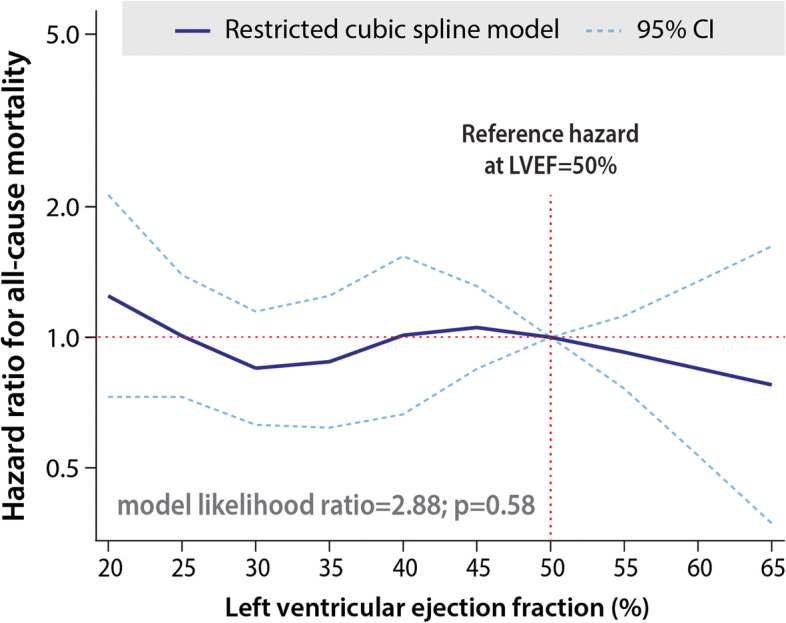
Association of ejection fraction with all-cause mortality. Spline analysis across the distribution of LVEF showing no overall association with all-cause mortality. Hazard of death is displayed in reference to patients with LVEF of 50%. LVEF, left ventricular ejection fraction

Factors independently associated with all-cause mortality in the multivariate model were limited to the presence of coronary artery disease (HR 2.34, 95% CI 1.77–3.08; *p* < 0.001) and hypertension (HR 1.45, 95% CI 1.11–1.88; *p* = 0.006); Additional file 1: Table S4. Category of HF (according to LVEF) and type of AF (according to temporal pattern) were not significantly associated with all-cause mortality; Fig. 1. LVEF was not related to all-cause mortality overall (HR 1.00 per 1% increase, 95% CI 0.98–1.01; *p* = 0.44). Figure 2 graphically depicts this lack of relationship between LVEF and all-cause mortality across the range of LVEF observed in study participants.

### Cause-specific mortality

**Fig. 3:**
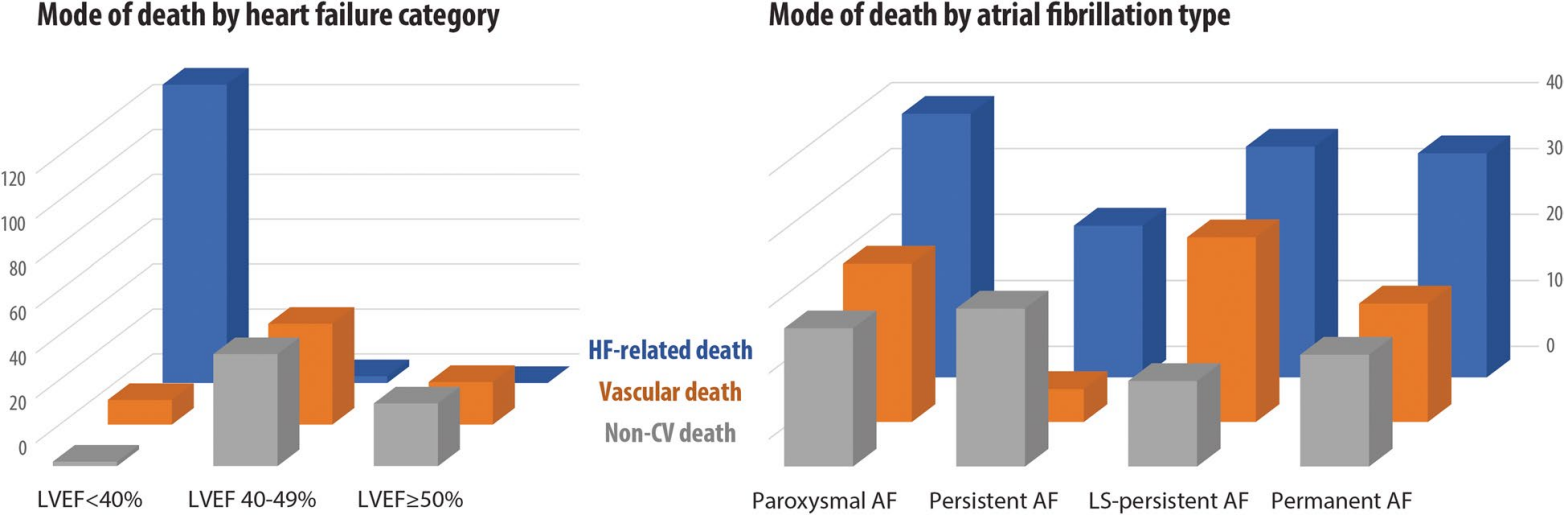
Modes of death by HF category and AF type. Number of deaths stratified by mode of death. The category of HF (left) is based on the LVEF assessment. The type of AF (right) is based on clinical assessment and excludes 33 patients with new-onset AF. AF, atrial fibrillation; CV, cardiovascular; HF, heart failure; LS, long-standing; LVEF, left ventricular ejection fraction

A full list of causes of death is presented in Table 2, including the differences between patients that underwent post-mortem examination. The 291 deaths were classified as HF-related in 136 patients (46.7%), vascular death in 75 patients (25.8%) and non-cardiovascular death in 80 patients (27.5%). The mode of death according to the HF category and AF type is presented in Fig. 3. Baseline variables associated with the different modes of death were unique and contrary, particularly with regard to LVEF (Table 3).

**Table 2:** Post-mortem and non-post-mortem causes of death

Mode of death	Post-mortem performed (*n* = 186)	Post-mortem not performed (*n* = 105)
HF-related death	Deaths = 92 (49.5%)	Deaths = 44 (41.9%)
	Dilated cardiomyopathy (*n* = 35)	Dilated cardiomyopathy (*n* = 17)
	Multi-organ congestion on post-mortem (*n* = 14)	Valvular heart disease (*n* = 7)
	Valvular heart disease (*n* = 12)	Hypertensive cardiomyopathy (*n* = 5)
	Myo-pericardial disease (*n* = 11)	Myo-pericardial disease (*n* = 4)
	Cardiac dystrophy due to brown atrophy (*n* = 9)	Hypertrophic cardiomyopathy (*n* = 2)
	Restrictive cardiomyopathy (*n* = 2)	Endocrine-related cardiomyopathy (*n* = 2)
	Hypertensive cardiomyopathy (*n* = 4)	Cardiomyopathy with an underlying systemic autoimmune condition (*n* = 2)
	Hypertrophic cardiomyopathy (*n* = 3)	Cardiomyopathy related to cancer treatment (*n* = 2)
	Cardiomyopathy with an underlying systemic autoimmune condition (*n* = 1)	Cardiomyopathy related to neuromuscular conditions (*n* = 1)
	Cardiomyopathy related to neuromuscular conditions (*n* = 1)	Restrictive cardiomyopathy (*n* = 1)
		Tako-Tsubo cardiomyopathy (*n* = 1)
Vascular death	Deaths = 41 (22.0%)	Deaths = 34 (32.4%)
	Myocardial infarction (*n* = 13)	Stroke (*n* = 16)
	Non-coronary/non-cerebral atherosclerosis (*n* = 11)	Non-coronary/non-cerebral atherosclerosis (*n* = 7)
	Stroke (*n* = 11)	Pulmonary embolism (*n* = 6)
	Pulmonary embolism (*n* = 4)	Myocardial infarction (*n* = 5)
	Aortic dissection (*n* = 2)	
Non-CV death	Deaths = 53 (28.5%)	Deaths = 27 (25.7%)
	Malignancies (*n* = 19)	Kidney failure (*n* = 10)
	Haemorrhage (*n* = 6)	Decompensated diabetes (*n* = 9)
	Pneumonia (*n* = 5)	Malignancies (*n* = 7)
	Kidney failure (*n* = 5)	Pneumonia (*n* = 1)
	Decompensated diabetes (*n* = 5)	
	Hypovolemic shock (*n* = 4)^a^	
	Endocarditis (*n* = 3)	
	Bronchopneumonia (*n* = 3)	
	COPD (*n* = 2)	
	Sepsis (*n* = 1)	

Percentages are the proportion of that mode of death in the post-mortem/non-post-mortem group (see Additional file 1: Online Methods for further details of the post-mortem process)

*COPD* chronic obstructive pulmonary disease, *CV* cardiovascular, *HF* heart failure

^a^The main cause of the hypovolemic shock was bleeding from ruptured oesophageal varices (three patients) and duodenal peptic ulceration (one patient)

**Table 3:** Comparison of factors associated with different modes of death

Multivariate analysis	Mode of death								
	**HF-related death**			**Vascular related death**			**Non-CV death**		
	**HR**	**95% CI**	***p*-value**	**HR**	**95% CI**	***p*-value**	**HR**	**95% CI**	***p*-value**
Age (per 1-year increase)	1.00	0.97–1.02	0.76	0.99	0.96–1.02	0.42	1.02	0.99–1.06	0.17
Gender (women vs men)	1.04	0.71–1.54	0.83	0.88	0.52–1.48	0.64	1.08	0.64–1.80	0.78
NYHA class (per 1 class increase)	**2.45**	**1.73–3.46**	** < 0.001**	0.82	0.61–1.10	0.19	0.76	0.56–1.02	0.07
LVEF (per 1% increase)	**0.95**	**0.93–0.97**	** < 0.001**	**1.08**	**1.05–1.11**	** < 0.001**	**1.10**	**1.06–1.13**	** < 0.001**
AF type (non-paroxysmal vs paroxysmal)	1.00	0.62–1.60	0.99	1.06	0.57–1.99	0.85	1.60	0.91–2.82	0.10
Clinical obesity (yes vs no)	1.46	0.94–2.26	0.09	0.83	0.44–1.57	0.57	**2.20**	**1.21–4.00**	**0.010**
Coronary artery disease (yes vs no)	0.82	0.50–1.37	0.46	1.05	0.55–2.02	0.87	0.82	0.46–1.45	0.49
Hypertension (yes vs no)	1.04	0.65–1.67	0.86	**2.83**	**1.36–5.90**	**0.005**	0.84	0.49–1.45	0.53
COPD (yes vs no)	0.85	0.55–1.31	0.46	1.17	0.65–2.13	0.60	0.85	0.49–1.47	0.56
Diabetes mellitus (yes vs no)	0.86	0.56–1.32	0.48	^a^			1.33	0.78–2.26	0.29

Hazards for each covariate presented are for that particular mode of death compared to other modes. All models are also adjusted for statins, renin–angiotensin–aldosterone antagonists, beta-blockers, diuretics, amiodarone and digoxin at baseline (not shown)

*AF* atrial fibrillation, *COPD* chronic obstructive pulmonary disease, *CV* cardiovascular, *HR* hazard ratio, *LVEF* left ventricular ejection fraction, *NYHA* New York Heart Association

^a^Not presented due to significant 3-way interaction with coronary artery disease and use of beta-blockers at baseline

Comparing HF-related death with the other causes of death, multivariate analysis identified higher NYHA class (HR 2.45 per one class increase, 95% CI 1.73–3.46; *p* < 0.001) and lower LVEF (HR 0.95 per 1% increase, 0.93–0.97; *p* < 0.001) as independently associated with HF-related death. The median NTproBNP level in this group of 11,869 pg/mL (IQR 5498–25,410) was significantly higher than for other causes of death, but with a broad range (*p* < 0.0001; Additional file 1: Fig. S2).

Factors associated with vascular death compared to other causes were hypertension (HR 2.83, 1.36–5.90; *p* = 0.005) and more preserved LVEF (HR 1.08 per 1% increase, 1.05–1.11; *p* < 0.001). The median NTproBNP level in this group was 7098 pg/mL (IQR 5054–9841).

Non-cardiovascular death compared to other causes identified obesity (HR 2.20, 1.21–4.00; *p* = 0.010) and more preserved LVEF (HR 1.10 per 1% increase, 1.06–1.13; *p* < 0.001) as significant predictors. The median NTproBNP level in this group of 6618 pg/mL (IQR 2029–9767) was not significantly different compared to patients with vascular death (*p* = 0.08).

Each mode of death had recognizable comorbidities and characteristics associated with them (Additional file 1: Table S5). However, the overall multi-morbidity burden (as estimated by clinical risk scores) was similar across those alive and the various modes of death. The mean baseline CHA_2_DS_2_-VASC risk score for stroke and thromboembolism was 5.2 ± 1.3 for patients subsequently alive at the end of follow-up, 5.0 ± 1.2 for HF-related death, 5.4 ± 1.4 for vascular death, and 5.1 ± 1.3 for non-CV death (*p* = 0.15). Similarly, HAS-BLED was similar across the groups: 3.1 ± 1.4, 2.9 ± 1.6, 3.4 ± 1.6 and 2.8 ± 1.4.

## Discussion

Uniquely supported by post-mortem examinations for in-hospital or unclear causes of death, this analysis of anticoagulated patients with HF and AF was able to differentiate clinical factors associated with specific modes of death. We identified that neither HF type nor AF type was independently associated with all-cause mortality, and the value of NTproBNP and LVEF assessment to predict the cause of death were limited. The extremely poor prognosis demonstrated in this population with both HF and AF highlights the need for a clear focus on the prevention of mortality and improvements in the management for these patients beyond anticoagulation.

There is a paucity of studies that report and assess post-mortem data in a patient with HF, AF or both. In 232 autopsies in patients with HF, discrepancies between clinical and post-mortem diagnoses were seen in 191 (82%) of cases, with major discrepancies with potential clinical impact in 91 (39%) [8]. By far, the most common mode and underlying cause of death in our patient cohort were connected to HF itself. Our finding, that nearly half of all deaths were related to HF, is consistent with prior data [6–8] and presents a key challenge for clinicians, especially as only LVEF and NYHA class were independent predictors of HF-related death. Ensuring optimal management according to the guidelines is vital to preventing excess mortality, improving LVEF and reducing symptoms [14]. In HFrEF, angiotensin receptor-neprilysin inhibitors are effective even in the context of AF [15], although other therapeutic strategies (e.g. resynchronization therapy) have lower efficacy when both conditions combine [16]. Beta-blockers are well established in HFrEF with sinus rhythm [17] where they clearly improve LVEF and NYHA class, but analysis of double-blind trials has questioned their efficacy in patients with AF [18]. Apart from recent data on gliflozins, we lack other therapies in clinical practice with known prognostic benefits for patients with HFpEF, and the value of improving LVEF is likely limited. However, symptom class may be amenable to treatment, including for patients with concomitant AF. For example, compared to beta-blockers, the use of low-dose digoxin leads to significant improvements in symptom class with significantly lower NTproBNP and adverse events [19]. Although physical-related quality of life was no different, patients randomized to low-dose digoxin had substantially better symptom control than beta-blockers: mean NYHA class 2.4 ± 0.5 at baseline improving to 1.5 ± 0.6 at 12 months versus 2.4 ± 0.6 to 2.0 ± 0.6 for beta-blockers (*p* < 0.001) [20].

Vascular causes accounted for a quarter of all the deaths in this cohort and were more common amongst those with preserved LVEF and hypertension. Although elevated systolic blood pressure is well recognized in the pathogenesis of HF and the sequalae of AF, relatively little attention has been paid to the management of hypertension in these conditions, and we lack specific randomized trials that demonstrate prognostic benefit. In a meta-analysis of 37 trials that assessed drugs with blood pressure-lowering properties, a small but significant decrease in systolic blood pressure was noted in patients with HF; however, there was no apparent association between the magnitude of blood pressure-lowering and cardiovascular events [21]. Conversely, in AF, there is a 9% reduction in the hazard of major cardiovascular events per 5 mmHg reduction in systolic blood pressure, identical to that observed for patients without AF [22]. Despite this, almost a quarter of AF patients have uncontrolled hypertension, as noted in a registry that spans 176 clinics across the USA, highlighting potential avenues to reduce vascular deaths in patients with HF and AF. Closely linked with uncontrolled hypertension in AF are deaths following acute stroke, which predominantly occurs in those who have not received anticoagulation. Although the residual risk of stroke and systemic embolus in anticoagulated patients is higher in those with concomitant HF, the absolute risk from trial data is small at 0.9 per 100 patient-years in those with non-permanent AF and 1.3 per 100 patient-years with permanent AF [23]. These data support the almost universal application of anticoagulation in this multimorbid population, but also the need to look beyond anticoagulation, particularly when targeting mortality reduction.

Non-cardiovascular death was numerically more common than vascular causes, consistent with the change in patient demographics seen over recent years. In a cohort of 57,818 new AF patients in primary care in the UK, rates of ischemic heart disease dropped from 44.1% in 1998–2001 to 37.3% by 2007–2010, whilst diabetes increased from 8.4 to 13.5% [24]. In heart failure, a longitudinal study of > 4 million patients demonstrated a rising number of comorbidities, increasing from 68% with 3 or more conditions in 2014 to 87% by 2014 [25]. We identified obesity as a significant independent predictor of non-cardiovascular death. The relationship between body mass index and mortality is complex, with the so-called ‘obesity paradox’ in HF likely due to a combination of induced selection biases (collider stratification bias) and other confounders [26, 27]. Our results would suggest weight loss could be valuable in patients with HF and AF to reduce the risk of non-cardiovascular death, consistent with previously identified benefits in other patient groups [28, 29]. Importantly, we confirmed that the management of coronary artery disease and hypertension should be a key focus in patients with HF and AF to prevent all-cause mortality, in line with current clinical consensus [30].

### Strengths and limitations

The patients included in this study were predominantly of European descent, with highly symptomatic HF and AF at presentation. Patients came from a wide geographic area including around half from rural communities. Due to the study’s aim of assessing post-mortem evidence where possible, a retrospective design was required. However, this design, and the inherent observational nature of the study, can lead to the potential selection and ascertainment biases. To reduce bias, we enrolled consecutive patients and ensured that data completion rates were high with no need for imputation and no patients had missing information on their vital status. We did not assess follow-up therapy, so it is possible that some patients withdrew or were withdrawn from their anticoagulant therapy after discharge. Details on cardiac resynchronization therapy were not available, and only 49 (4.9%) had an implanted cardiac defibrillator as device implantation required referral to other hospitals.

A key strength of our analysis is the availability of post-mortem examinations for a large proportion of cases. The need for an autopsy was principally determined by the timing of death (within 24 h of admission). However, attending physicians were also able to request a post-mortem if the cause of death was not clear and the family did not object. We used the recorded cause of death to avoid any issues of competing diagnoses; however, in clinical practice, it is likely that other underlying conditions would also have played a role in the patient’s demise. We do not report on sudden cardiac deaths as these are rarely documented as causes of death within Romania. Pathologists instead record any underlying diseases contributing to mortality, which is a limitation to the generalizability of our study to non-hospitalized populations. Autopsies may have misrepresented deaths due to ventricular arrhythmia in cases with other incidental findings (e.g. occlusive coronary disease), although the clinical staff had full access to hospital records when ascertaining the cause of death. There is a possibility of a miscoded cause of death in those not undergoing post-mortem examination; however, we saw only small differences in the underlying aetiology, with fewer vascular and more HF-related deaths in those with autopsy information. Finally, any observations are limited by the number of death events; however, this was large in comparison with other published data with 3 in 10 patients dying during the study period.

## Conclusions

The extremely poor prognosis in this anticoagulated population with HF and AF highlights the need for further attention to reduce excess deaths. This includes better management of HF to reduce symptoms and increase LVEF (thereby preventing HF-related death), a clear focus on control of hypertension (to prevent vascular death) and tackling lifestyle factors such as obesity (contributing to the prevention of non-cardiovascular death). Supported by post-mortem examinations, this study demonstrates that further research is required on the cause of death in these patients, beyond the simple classification of the category of HF and type of AF.

## Supplementary Information

**Additional file 1:** Online Methods. **Table S1.** Definitions for heart failure. **Table S2.** Definitions for atrial fibrillation. **Table S3.** Baseline characteristics by post-mortem examination. **Table S4.** Factors associated with all-cause mortality. **Table S5.** Baseline characteristics by different modes of death. **Fig. S1.** Post-mortem histology examples. **Fig. S2.** NT-pro B-type natriuretic peptide values on admission for deceased patients.

**Additional file 2.** CODE-EHR minimum standards framework reporting checklist (use of health record data for clinical research).

## Data Availability

Individual patient data are not available for this study; all relevant analyses are available on request to work with anonymized datasets.

## Abbreviations

AF: Atrial fibrillation
CHA_2_DS_2_-VAS_C_: Risk score for thromboembolism in AF
HAS-BLED: Risk score for bleeding in AF
HF: Heart failure
HFpEF: Heart failure with preserved ejection fraction
HFrEF: Heart failure with reduced ejection fraction
LVEF: Left ventricular ejection fraction
NTproBNP: N-terminal prohormone B-type natriuretic peptide
NYHA: New York Heart Association
