# Supplementary material for: Post-mortem examination of high mortality in patients with heart failure and atrial fibrillation
Source: BMC Med. 2022 Oct 5;20:331. doi: 10.1186/s12916-022-02533-8 (PMC9533594; doi:10.1186/s12916-022-02533-8)
Supplement: Supplementary file 1 — Additional file 1: Online Methods. Table S1. Definitions for heart failure. Table S2. Definitions for atrial fibrillation. Table S3. Baseline characteristics by post-mortem examination. Table S4. Factors associated with all-cause mortality. Table S5. Baseline characteristics by different modes of death. Fig. S1. Post-mortem histology examples. Fig. S2. NT-pro B-type natriuretic peptide values on admission for deceased patients. [file 12916_2022_2533_MOESM1_ESM.docx]

**Post-mortem examination of high mortality in patients with heart failure and atrial fibrillation**

Otilia Țica, Ovidiu Țica, Karina V Bunting, Joseph deBono, Georgios V Gkoutos, Mircea I. Popescu, Dipak Kotecha.

**Additional file 1**

[ONLINE METHODS 2](#_Toc111465253)

[Dataset construction and linkage 2](#_Toc111465254)

[Data fit for purpose 2](#_Toc111465255)

[Disease and outcome definitions 3](#_Toc111465256)

[Analysis 4](#_Toc111465257)

[Ethics and governance 4](#_Toc111465258)

[Table S1: Definitions for heart failure 6](#_Toc111465259)

[Table S2: Definitions for atrial fibrillation 7](#_Toc111465260)

[Table S3: Baseline characteristics by post-mortem examination 8](#_Toc111465261)

[Table S4: Factors associated with all-cause mortality 9](#_Toc111465262)

[Table S5: Baseline characteristics by different modes of death 10](#_Toc111465263)

[Figure S1: Post-mortem histology examples 11](#_Toc111465264)

[Figure S2: NTproBNP values on admission for deceased patients 12](#_Toc111465265)

# ONLINE METHODS

This section details methodology of the study for full transparency, and to meet the best practice framework for the use of electronic healthcare records in clinical research (CODE-EHR) [10] and the Strengthening the Reporting of Observational Studies in Epidemiology (STROBE) Statement. The following sections address each item of the CODE-EHR framework; see **Additional file 2** for details of each domain.

## Dataset construction and linkage

1. Source of data: Study data were sourced from the Emergency County Clinical Hospital of Oradea (SCJUO), Romania, InfoWord database, patient medical chart, Integrated Single Computer System (SIUI) and from the death certificate registry. SIUI is the National Health Insurance House portal, which includes the daily work tool of doctors and nurses who update information in this system in real time and offline (<http://siui.casan.ro/cnas/>), linking together and coordinating the Romanian social health insurance system. Human input error is minimised by crosslinking with other national databases, such as Population Records, the National Agency for Pensions and Social Insurance, and the National Employment Agency.
2. Approach to missing data: In this study, only patients with complete case data were used in regression analysis, with no imputation performed.
3. Completeness of follow-up: The SIUI system includes all deaths across Romania.
4. Linkage: Datasets were unified within the hospital domain, with patients in this study matched using internal hospital identifiers.

## Data fit for purpose

1. Origin, process and purpose of data: Attending physicians have the obligation and responsibility to complete and codify the main diagnosis and the secondary diagnoses, noting all clinical care at discharge.
2. Coding systems: The International Statistical Classification of Diseases and Related Health Problems – Tenth Revision (ICD-10) coding scheme with Australian Amendments (CIM-10-AM) applies to both public and private hospitals in Romania.
3. Quality assessment: Clinical staff compiling the dataset made checks for legitimacy, and cross-checked across each medical record for consistency. No external quality assessments were performed.
4. Potential sources of bias: None known. No financial incentives were involved for the attending physician.

## Disease and outcome definitions

1. Disease definitions: Apart from comorbidities specifically discussed in the main methods section, the definitions of diseases were accepted as recorded by the attending physician(s). HF and AF were classified as per **Additional file 1: Table S1** and **Additional file 1: Table S2** below. See main methods section on assessment of mortality. Definitions were agreed prior to statistical analysis.
2. Coding manual: Not applicable.
3. Phenotyping approach: Post-mortem examinations were performed according to a standardised protocol. In-hospital deaths need to be followed by a post-mortem with only few exceptions stated by Romanian law. The autopsy technique is standardised, and training and performed systematically according to guidelines issued by Romanian Ministry of Health. Following full external assessment, evisceration is performed after a minimum of 6 hours from death, mainly using the Rokitansky method that maintains anatomical relations. Other techniques can be used in different situations. Heart examination is performed mainly using atrio-ventricular opening on both sides which facilitate valve measurements, proper evaluation of cusps and the dissection of anterior and posterolateral myocardial wall. Harvesting specimens for histology is mandatory for completion of the post-mortem report.
4. Validation of coding: As internationally accepted definitions for HF and AF were used, no additional validation was performed.

## Analysis

1. Statistical methods: Please see main paper methods section.
2. Machine code: No machine code or algorithms were used in the analysis.
3. Internal validation: Multivariate Cox models were repeated to include a broader range of variables using stepwise approaches, without major impact of study findings. Effect modification was assessed using p values from interaction terms fitted in the multivariable models. The interaction of continuous LVEF with mortality was assessed using cubic splines in the Cox model – this approach is a form of generalized linear model useful for assessing continuous variables. The analysis used 5 knots with LVEF ranging from 20 to 65% in 5% increments, and a reference LVEF of 50%. Proportional hazards assumptions for all-cause mortality were confirmed using Schoenfeld residuals. A statistical analysis plan has not been previously published.
4. External validation: This was not possible due to the requirement for post-mortem examination.

## Ethics and governance

1. Consent: The study was conducted in accordance with the ethical principles set out in the Helsinki Declaration and Recommendations for Good Clinical Practice, and was approved by the SCJUO Ethics Committee (32926/2017) without the need for individual patient consent.
2. Data privacy: All patient data were pseudonymised and given a unique alphanumeric code after extraction from clinical systems by the team in Romania. Full anonymisation of data occurred prior to statistical analysis at the University of Birmingham.
3. Patient and public involvement: Not performed as part of the study design or management. PPI provided for evaluation and dissemination as part of the card*AI*c Clinical & Data Science Forum at the University of Birmingham/University Hospitals Birmingham NHS Foundation Trust, UK.
4. Data sharing: Anonymised data are available under reasonable request by contacting the corresponding author(s). Source data can be verified, but only by staff with relevant clinical need at SCJUO.

# Table S1: Definitions for heart failure

| **Type of HF** | **Classification** | **Definition according to ESC guidelines** | **Clinical diagnosis** |
| --- | --- | --- | --- |
| Based on LVEF | HF with reduced LVEF (HFrEF); LVEF <40% | HF is a clinical syndrome characterized by typical symptoms (e.g. breathlessness, ankle swelling and fatigue) that may be accompanied by signs (e.g. elevated jugular venous pressure, pulmonary crackles and peripheral oedema) caused by a structural and/or functional cardiac abnormality, resulting in a reduced cardiac output and/or elevated intracardiac pressures at rest or during stress.[11] | Signs and symptoms of left ventricular failure (exertional dyspnoea, cough, fatigue, orthopnoea, paroxysmal nocturnal dyspnoea, cardiac enlargement, rales, gallop rhythm, pulmonary venous congestion).  Confirmed by supporting evidence from non-invasive or invasive measurements of systolic and/or diastolic dysfunction. |
|  | HF with mid-range/mildly reduced LVEF (HFmrEF); LVEF 40-49% |  |  |
|  | HF with preserved LVEF (HFpEF); LVEF ≥50% |  |  |
| Based on NYHA class | NYHA class I: No limitation of physical activity. Ordinary physical activity does not cause undue fatigue, palpitation or dyspnoea. |  |  |
|  | NYHA class II: Slight limitation of physical activity. Comfortable at rest. Ordinary physical activity results in fatigue, palpitation or dyspnoea. |  |  |
|  | NYHA class III: Marked limitation of physical activity. Comfortable at rest. Less than ordinary activity causes fatigue, palpitation, or dyspnoea. |  |  |
|  | NYHA Class IV: Unable to carry on any physical activity without discomfort. Symptoms of heart failure at rest. If any physical activity is undertaken, discomfort increases. |  |  |

ESC = European Society of Cardiology; HF = heart failure; LVEF = left ventricular ejection fraction; NYHA = New York Heart Association.

# Table S2: Definitions for atrial fibrillation

| **Type of AF** | **Classification** | **Definition according to ESC guidelines** | **Clinical diagnosis** |
| --- | --- | --- | --- |
| First episode of AF | AF that has not been diagnosed before, irrespective of the duration of the arrhythmia or the presence and severity of AF-related symptoms. | The diagnosis of AF requires rhythm documentation using an ECG showing the typical pattern of AF: Irregular RR intervals and no discernible, distinct P waves.  By accepted convention, an episode lasting at least 30 seconds is diagnostic.[12] | 12-lead ECG or ambulatory ECG recording showing AF in patient’s history or during hospitalisation. |
| Paroxysmal AF | Self-terminating, in most cases within 48 hours. Some AF paroxysms may continue for up to 7 days. AF episodes that are cardioverted within 7 days should be considered paroxysmal. |  |  |
| Persistent AF | AF that lasts longer than 7 days, including episodes that are terminated by cardioversion, either with drugs or by direct current cardioversion, after 7 days or more. |  |  |
| Long standing persistent AF | Continuous AF lasting for ≥ 1year when it is decided to adopt a rhythm control strategy. |  |  |
| Permanent AF | AF that is accepted by the patient (and physician). Hence, rhythm control interventions are, by definition, nor pursued in these patients. Should a rhythm control strategy be adopted, the arrhythmia would be re-classified as ‘long-standing persistent AF’. |  |  |

AF = atrial fibrillation; ECG = electrocardiogram; EHRA = European Heart Rhythm Association; ESC = European Society of Cardiology.

# Table S3: Baseline characteristics by post-mortem examination

| Characteristic | | Post-mortem performed  (n=186) | Post-mortem not performed  (n=105) | p-value |
| --- | --- | --- | --- | --- |
| Age, mean % ±SD | | 72.9±9.2 | 73.9±8.8 | 0.73 |
| Women, n (%) | | 79 (42.5%) | 54 (51.4%) | 0.14 |
| Background (urban vs rural setting), n (%) | | 75 (40.3%) | 58 (55.2%) | 0.014* |
| NYHA class | Mean ±SD | 3.0±0.9 | 3.0±0.9 | 0.52 |
|  | class I, n (%) | 18 (9.7%) | 5 (4.8%) |  |
|  | class II, n (%) | 34 (18.3%) | 19 (18.1%) |  |
|  | class III, n (%) | 67 (36.0%) | 43 (41.0%) |  |
|  | class IV, n (%) | 67 (36.0%) | 38 (36.2%) |  |
| LVEF | Mean % ±SD | 39.3±10.8 | 41.2±11.2 | 0.11 |
|  | <40%, n (%) | 101 (54.3%) | 45 (42.9%) |  |
|  | 40-49%, n (%) | 57 (30.6%) | 41 (39.1%) |  |
|  | ≥50%, n (%) | 28 (15.1%) | 19 (18.1%) |  |
| AF type, n (%) | new onset, n (%) | 9 (4.9%) | 0 (0.0%) | 0.07 |
|  | paroxysmal, n (%) | 54 (30.5%) | 31 (29.5%) |  |
|  | persistent, n (%) | 41 (23.1%) | 11 (10.5%) |  |
|  | long standing persistent, n (%) | 46 (26.0%) | 30 (28.6%) |  |
|  | permanent, n (%) | 36 (20.3%) | 33 (31.4%) |  |
| Coronary artery disease, n (%) | | 144 (77.4%) | 69 (65.7%) | 0.031 |
| Hypertension, n (%) | | 144 (77.4%) | 60 (57.1%) | <0.001 |
| Chronic obstructive pulmonary disease, n (%) | | 62 (33.3%) | 26 (24.8%) | 0.12 |
| Diabetes mellitus, n (%) | | 65 (35.0%) | 33 (31.4%) | 0.54 |
| Chronic kidney disease, n (%) | | 60 (32.3%) | 34 (32.4%) | 0.98 |
| Prior stroke or transient ischaemic attack, n (%) | | 19 (10.2%) | 26 (24.8%) | 0.019 |
| Obesity (clinical diagnosis), n (%) | | 72 (38.7%) | 20 (19.1%) | 0.002 |
| CHA_2_DS_2_ -VAS_C_ score, mean ±SD | | 5.0±1.3 | 5.4±1.4 | 0.05 |
| HAS-BLED score, mean ±SD | | 2.6±1.3 | 3.7±1.9 | 0.004 |
| ACE inhibitor or ARB, n (%) | | 148 (79.6%) | 77 (73.3%) | 0.22 |
| Beta-blockers, n (%) | | 109 (58.6%) | 67 (63.8%) | 0.38 |
| Diuretics or MRA, n (%) | | 157 (84.4%) | 85 (81.0%) | 0.45 |
| Amiodarone, n (%) | | 89 (47.9%) | 22 (21.0%) | 0.006 |

* no longer significant when accounting for multiple testing.

ACEi = angiotensin-converting enzyme inhibitors; AF = atrial fibrillation; ARB = angiotensin receptor blocker; CHA_2_DS_2_ -VAS_C_ = risk score for thromboembolism in AF; HAS-BLED = risk score for bleeding in AF; IQR = interquartile range; LVEF = left ventricular ejection fraction; MRA = mineralocorticoid receptor antagonist; NYHA = New York Heart Association; SD = standard deviation.

# Table S4: Factors associated with all-cause mortality

| **Multivariate analysis** | **Hazard ratio** | **95% CI** | **p-value** |
| --- | --- | --- | --- |
| Age (per 1 year increase) | 1.01 | 1.00 - 1.02 | 0.22 |
| Gender (women vs men) | 0.86 | 0.68 - 1.09 | 0.21 |
| NYHA class (per 1 class increase) | 1.02 | 0.88 - 1.19 | 0.77 |
| LVEF (per 1% increase) | 1.00 | 0.98 - 1.01 | 0.44 |
| AF type (non-paroxysmal vs paroxysmal) | 1.10 | 0.84 - 1.42 | 0.49 |
| Clinical obesity (yes vs no) | 1.13 | 0.87 - 1.46 | 0.36 |
| Coronary artery disease (yes vs no) | **2.34** | **1.77 – 3.08** | **<0.001** |
| Hypertension (yes vs no) | **1.45** | **1.11 - 1.88** | **0.006** |
| COPD (yes vs no) | 0.85 | 0.66 - 1.10 | 0.21 |
| Diabetes mellitus (yes vs no) | 1.13 | 0.88 - 1.45 | 0.34 |

All models are also adjusted for statins, renin-angiotensin-aldosterone antagonists, beta-blockers, diuretics, amiodarone and digoxin at baseline (not shown).

AF = atrial fibrillation; COPD = chronic obstructive pulmonary disease; LVEF = Left ventricular ejection fraction; NYHA = New York Heart Association.

Table S5: Baseline characteristics by different modes of death

| Characteristic | | Alive at end of follow-up  (n=718) | HF-related death  (n=136) | Vascular death  (n=75) | Non-CV death  (n=80) |
| --- | --- | --- | --- | --- | --- |
| Age, mean % ±SD | | 72.6±11.0 | 73.4±8.4 | 72.8±11 | 73.4±8.2 |
| Women, n (%) | | 343 (47.8%) | 63 (46.3%) | 32 (42.7%) | 38 (47.5%) |
| Background (urban vs rural setting), n (%) | | 370 (51.5%) | 53 (39.0%) | 35 (46.7%) | 45 (56.3%) |
| NYHA class | Mean ±SD | 3.0±0.9 | 3.7±0.6 | 2.5±0.9 | 2.4±0.7 |
| LVEF | Mean % ±SD | 40.1±11.1 | 30.4±6.2 | 47.2±7.1 | 49.5±5.6 |
|  | <40%, n (%) | 341 (47.5%) | 133 (98.0%) | 11 (14.7%) | 2 (2.5%) |
|  | 40-49%, n (%) | 244 (34.0%) | 3 (2.2%) | 45 (60.0%) | 50 (62.5%) |
|  | ≥50%, n (%) | 133 (18.5%) | 0 (0.0%) | 19 (25.3%) | 28 (35.0%) |
| AF type, n (%) | new onset, n (%) | 24 (3.3%) | 4 (3.0%) | 0 (0.0%) | 5 (6.3%) |
|  | paroxysmal, n (%) | 202 (29.1%) | 40 (30.3%) | 24 (32.0%) | 21 (28.0%) |
|  | persistent, n (%) | 122 (17.6%) | 23 (17.4%) | 5 (6.7%) | 24 (32.0%) |
|  | long standing persistent, n (%) | 178 (25.6%) | 35 (26.6%) | 28 (37.3%) | 13 (17.3%) |
|  | permanent, n (%) | 192 (27.7%) | 34 (25.8%) | 18 (24.0%) | 17 (22.7%) |
| Coronary artery disease, n (%) | | 331 (46.1%) | 98 (72.1%) | 60 (80.0%) | 55 (68.8%) |
| Hypertension, n (%) | | 378 (52.6%) | 89 (65.4%) | 65 (86.7%) | 50 (62.5%) |
| Chronic obstructive pulmonary disease, n (%) | | 263 (36.6%) | 40 (29.4%) | 20 (26.7%) | 20 (35.0%) |
| Diabetes mellitus, n (%) | | 195 (27.2%) | 52 (38.2%) | 13 (17.3%) | 33 (41.2%) |
| Chronic kidney disease, n (%) | | 215 (29.9%) | 39 (28.7%) | 23 (30.7%) | 32 (40.0%) |
| Prior stroke or transient ischaemic attack, n (%) | | 116 (16.2%) | 14 (10.3%) | 28 (37.3%) | 3 (3.7%) |
| Obesity (clinical diagnosis), n (%) | | 175 (24.4%) | 39 (28.7%) | 23 (30.7%) | 30 (37.5%) |
| CHA_2_DS_2_ -VAS_C_ score, mean ±SD | | 5.2±1.3 | 5.0±1.2 | 5.4±1.4 | 5.1±1.3 |
| HAS-BLED score, mean ±SD | | 3.1±1.4 | 2.9±1.6 | 3.4±1.6 | 2.8±1.4 |

AF = atrial fibrillation; CHA_2_DS_2_ -VAS_C_ = risk score for thromboembolism in AF; HAS-BLED = risk score for bleeding in AF; LVEF = left ventricular ejection fraction; Non-CV = non-cardiovascular; NYHA = New York Heart Association; SD = standard deviation.

# Figure S1: Post-mortem histology examples


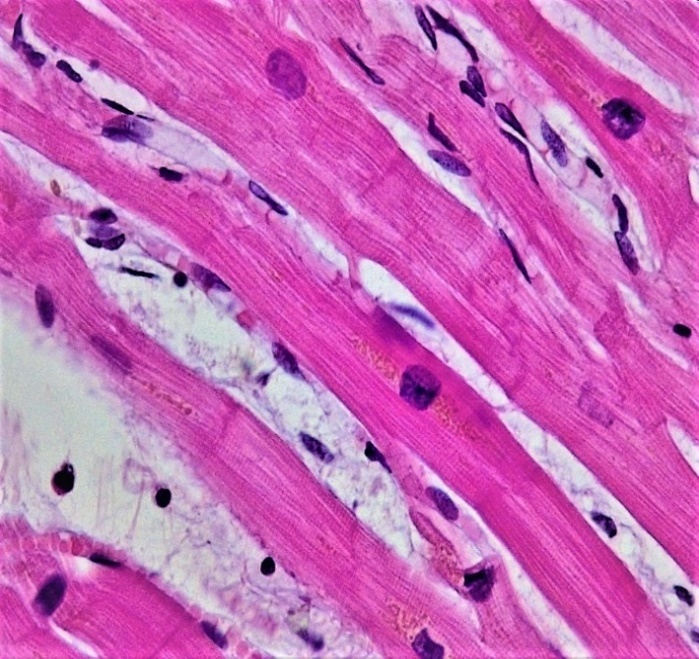

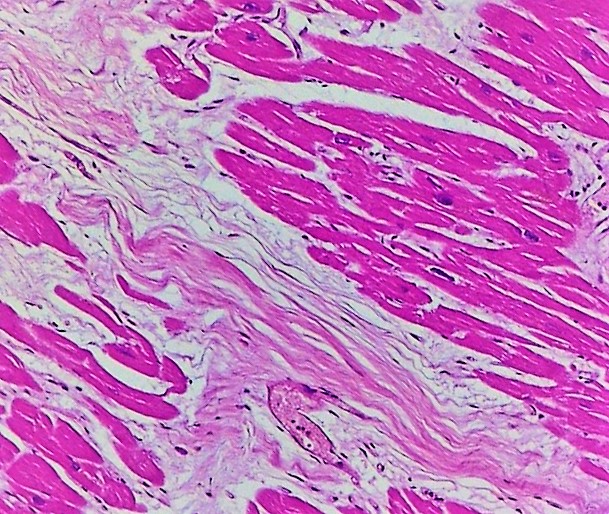


Histology samples from patients with heart failure and atrial fibrillation in the study undergoing post-mortem examination.

Left: Example of cardiac dystrophy due to brown atrophy; Haematoxylin-Eosin stain with 40X magnification. Myocardial fibres show single central squared nuclei and yellow-brown granules (lipofuscin) in the perinuclear area. The cytoplasm is uneven and the transversal striae are not constant. Some of the fibres are wavy and the intercalated disc are not evident. In the interstitial space, oedema and lymphocytes are present.

Right: Example of coronary atherosclerosis; Haematoxylin-Eosin stain with 10X magnification. Star-like perivascular fibrosis is noted, composed mainly of collagen fibres, fibroblasts and fibrocytes. The cardiomyocytes have eosinophilic cytoplasm and some of them are missing the nucleus secondary to chronic ischemia.

# Figure S2: NTproBNP values on admission for deceased patients


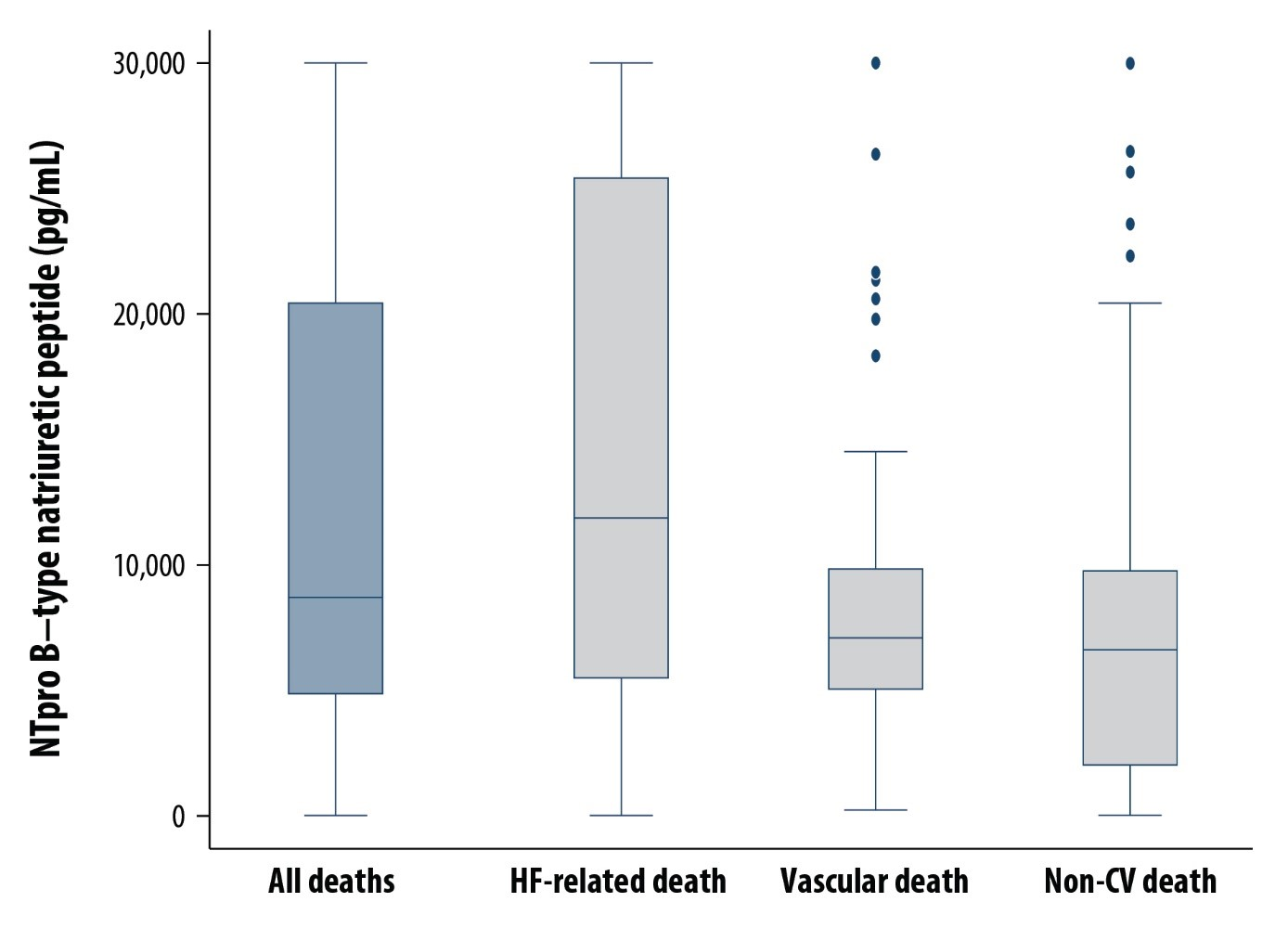


Box and whisker plot for baseline NTproBNP levels in those dying during the course of the study (n=291), subdivided by mode of death. Dots represent outside values. Patients with HF-related death had significantly higher NTproBNP compared to vascular death (p<0.0001) or non-CV death (p<0.0001). These remained significant after adjustment for multiple testing. There was no significant difference in NTproBNP levels comparing those dying of vascular and non-CV causes (p=0.08).

CV = cardiovascular; HF = heart failure; NTproBNP = N-terminal pro B-type natriuretic peptide.
